# Prediction of Dengue Incidence in the Northeast Malaysia Based on Weather Data Using the Generalized Additive Model

**DOI:** 10.1155/2021/3540964

**Published:** 2021-10-25

**Authors:** Afiqah Syamimi Masrani, Nik Rosmawati Nik Husain, Kamarul Imran Musa, Ahmad Syaarani Yasin

**Affiliations:** ^1^Department of Community Medicine, Universiti Sains Malaysia, Malaysia; ^2^Vector Unit, Kelantan State Health Department, Malaysia

## Abstract

**Introduction:**

Dengue, a vector-borne viral illness, shows worldwide widening spatial distribution beyond its point of origination, namely, the tropical belt. The persistent hyperendemicity in Malaysia has resulted in the formation of the dengue early warning system. However, weather variables are yet to be fully utilized for prevention and control activities, particularly in east-coast peninsular Malaysia where limited studies have been conducted. We aim to provide a time-based estimate of possible dengue incidence increase following weather-related changes, thereby highlighting potential dengue outbreaks.

**Method:**

All serologically confirmed dengue patients in Kelantan, a northeastern state in Malaysia, registered in the eDengue system with an onset of disease from January 2016 to December 2018, were included in the study with the exclusion of duplicate entry. Using a generalized additive model, climate data collected from the Kota Bharu weather station (latitude 6°10′N, longitude 102°18′E) was analysed with dengue data.

**Result:**

A cyclical pattern of dengue cases was observed with annual peaks coinciding with the intermonsoon period. Our analysis reveals that maximum temperature, mean temperature, rainfall, and wind speed have a significant nonlinear effect on dengue cases in Kelantan. Our model can explain approximately 8.2% of dengue incidence variabilities.

**Conclusion:**

Weather variables affect nearly 10% of the dengue incidences in Northeast Malaysia, thereby making it a relevant variable to be included in a dengue early warning system. Interventions such as vector control activities targeting the intermonsoon period are recommended.

## 1. Introduction

Dengue, a vector-borne viral illness transmitted by the vector *Aedes* mosquito, is estimated to infect 390 million people globally each year [1]. In recent years, it has exhibited increasing worldwide spatial distribution involving 129 countries with a 20% case fatality rate, when left untreated [2]. Clinical manifestations of dengue range from asymptomatic infection or mild illness with constitutional symptoms to severe or life-threatening disease. With four distinct but closely related circulating dengue virus serotypes, life-long immunity developed against a specific infecting serotype upon recovery does not prevent infection by other serotypes. In contrast, subsequent infections by different serotypes could exacerbate the patient's risk of developing severe dengue [1]. This tropical climate favouring disease is endemic to the tropical belt of Asia, Latin America, Africa, and Australia. Locally, it has been observed to cause a cyclical pattern of three to five years in Malaysia, with all four dengue serotypes circulating concurrently. Recent studies in Malaysia have revealed that climate plays an integral role in affecting the magnitude of dengue incidences [3, 4]. The increase is secondary to the direct and indirect impact of weather on dengue transmission.

Temperature is also known to play a vital role in the transmission of dengue through affecting survivability and activities of the vectors, replicability of the agent, and behaviours of the host. Several studies have identified the mosquitos' feeding behaviour, oviposit activity, larva, and adult mosquito survivability to be temperature-dependent [5–7]. These mosquitos are reported to be most active between 26°C and 30°C for breeding activities [6] whilst their feeding decreases and mortality increases when the temperature is higher than 35°C or lower than 15°C [8]. The optimum temperature for the extrinsic incubation period (EIP), which refers to the infective cycle of the dengue virus where the virus replicates and migrates inside the body of the female *Aedes* mosquito, is between 25°C and 28°C [1].

The correlation of rainfall with dengue incidence shows mixed results. Aziz et al. [9] noted no significant correlation between monthly rainfall with the number of dengue cases. This result contradicts the findings from Rohani [10] and Dom et al. [11], which reveal a strong positive correlation between rainfall and dengue incidence. The difference in result could be attributed to the fact that both latter studies use vector density as an inference for dengue cases compared to the earlier research.

The linkage between wind speed and dengue incidences varies based on local variation and usually shows a nonlinear relation. A study by Mala and Jat [12] in Delhi indicates a positive association between increasing mean wind speed up to 3 km/h with a rise in dengue incidence. Cheong et al. [6] noted a similar finding but with a higher range of wind speed (between 5.5 km/h and 9.3 km/h) in their study in Putrajaya. This increase was ascribed to an increase in the mosquito flight range, which allows for a wider range of feeding. However, a stronger wind speed was noted to decrease the mosquito population density and reduce host-seeking activity [13]. Although Mala and Jat [12] did report a similar inverse relationship between stronger wind speed and dengue incidence, they also noted a wider spatial dispersion of dengue incidences due to strong wind speed.

Weather variables were also reported to be associated with dengue outbreaks. In Malaysia, a dengue outbreak occurs when two or more dengue cases are reported from the same locality within seven days. Evidence of the relationship between weather variables and dengue outbreak or spatial clustering of dengue, however, is relatively inconsistent in the literature. In Thailand, for example, urbanization, rather than rainfall or temperature, was associated with the spatial clustering of dengue [14]. Another study by Tian et al. [15] in Guangzhou, China, determines that increased surface water is linked to dengue outbreaks irrespective of the amount of rainfall in the area. However, Aziz et al. [9] reported in their study that the amount of rainfall has an association with spatial clustering or dispersion of dengue cases in Kuala Lumpur.

Different values in weather produce varying effects on vector distribution, survival, and activity, thus causing a complex, nonlinear relationship between weather and dengue cases [16]. Inadequate exploratory analysis of weather and dengue data leading to the usage of the classical linear modelling method may cause underfitting of the model [17]. Loss of this distinct nonlinear pattern may subsequently cause the inability to explain seasonal variations affecting dengue incidences, thereby making nonlinear modelling a better analytical choice [14]. The Poisson general additive model (GAM) is advantageous when identifying nonlinear relationships with an uncertain pattern of the relationship as it does not require any prior knowledge of the shape of the response curve [6].

Despite the hyperendemicity of dengue in Malaysia with periodic epidemics, proactive measures against the increase in dengue cases predicted by weather change have not been fully utilized. Some studies have been conducted to predict dengue occurrences through weather variables. However, these studies are location-specific and mainly focus on the west coast of peninsular Malaysia. To the best of our knowledge, only a limited number of studies have been conducted in Kelantan despite its being ranked first in the east coast of peninsular Malaysia and fourth in Malaysia, in terms of dengue cases in 2019. Thus, in this paper, we aim to provide a time-based prediction of possible dengue incidence increase following weather changes, highlighting potential dengue outbreaks.

## 2. Methods

This study was a retrospective secondary data review involving three years of aggregated quantitative data (2016 to 2018) from the eDengue system, Malaysia's dengue surveillance system. Permission to access the eDengue system was obtained from the Kelantan State Health Department with ethical approval from the National Medical Research Register. All serologically confirmed dengue cases registered in Kelantan with the onset of disease from 1^st^ January 2016 to 31^st^ December 2018 were included in the study whereas duplicate entries within the same incubation period of 14 days or imported cases where the dengue exposure occurred outside of Kelantan were excluded from the study. The weather data were obtained from the weather database of the Kota Bharu weather station (latitude 6°10′N, longitude 102°18′E) under the Malaysian Meteorological Department, which involves daily temperature, rainfall, and wind speed variables. Our study area is Kelantan, a state located on the east coast of peninsular Malaysia with the coordinates 5°15′N, 102°0′E. It spans an area of 17,100 km^2^ with a population of 1.89 million people in 2019.

The sample size was calculated with a two-tailed *z*-test formula for Poisson regression. The *α* was set at 0.05 and the power at 80%. *β*_0_, the baseline dengue incidence rate in Kelantan, is 0.024 [18], while *β*_1_ denotes the estimated changes of dengue cases in response to different weather variables. *R*^2^ signifies the possible interaction between covariates, of which we expected a moderate effect size of the weather variables hence set at 0.025. The mean exposure of the variable for one year is estimated at 0.5. An additional 10% was calculated to account for the possibility of missing or incomplete data. As the pooled data collected from eDengue for the stipulated period was smaller (10,645) than the highest calculated sample size required (10,704), all serologically confirmed dengue patients in Kelantan registered in the eDengue system with an onset of disease from January 2016 to December 2018 were included. On the other hand, the duplicate data of a confirmed dengue patient within two incubation periods of up to 14 days were excluded.

The daily count of dengue cases in the analysis was assumed to follow the Poisson distribution. The number of dengue cases was aggregated to a daily count producing 1,096 rows of data which were matched with the daily recorded weather variable. Each row corresponds to a single date within the three years. All five weather variables, minimum temperature, maximum temperature, mean temperature, rainfall, and wind speed, were initially included in the analysis. Correlation analysis was performed between weather variables and dengue cases. The minimum temperature was noted to have a high positive correlation with maximum temperature and mean temperature and was thus excluded from further analysis.

The effect of weather on dengue cases was evaluated by using the generalized additive model (GAM) function in the “mgcv” R package, version 1.8-31. Cubic smoothing function and *Poisson* family were applied. Forward and backward stepwise variable selection was used to construct the model based on the dispersion of the new estimated data around a *y* = *x* line (*R*^2^). The significance of the spline terms was assessed and fitted to the model. The best parsimonious model was selected based on the highest *R*^2^. We have identified the model with cubic regression splines for maximum temperature, mean temperature, rainfall, and wind speed as the best model (*R*^2^: 0.0818; deviance explained: 14.9%). The final model is depicted in the formula as follows:

(1) $$\begin{array}{c} \text{Dengue cases}\sim s\left(\text{maximum temperature},\text{bs}=\text{cr}\right)+s\left(\text{mean temperature},\text{bs}=\text{cr}\right)+s\left(\text{rainfall},\text{bs}=\text{cr}\right)+s\left(\text{windspeed},bs=\text{cr}\right). \end{array}$$

Note that *s* denotes the function used in the definition of smooth terms within a GAM formula, bs is the B spline which indicates the type of basis-penalty smoother, and cr is a penalized cubic regression spline defined by a modest-sized set of knots spread evenly throughout the values.

## 3. Results

A total of 10,645 serologically confirmed dengue cases were registered in Kelantan between 2016 and 2018, with a mean of 9.8 cases daily (SD = 11.02). The working population accounted for 69.9% of overall dengue incidence (mean age 28.8 years, SD = 1.23) with a nearly equal distribution between gender. Malays, who constituted most of the ethnic population in Kelantan, constituted the highest proportion (97.0%), followed by Chinese (2.1%) and others (0.9%) (Table 1).

Weather variables from the Kota Bharu weather station show readings consistent with tropical weather with little variability per day (Table 2) except for daily rainfall readings, where a wide standard deviation of 22.83 per day was observed. This pattern corresponds with the monsoon season affecting the east coast region of peninsular Malaysia between October and March.

**Figure 1:**
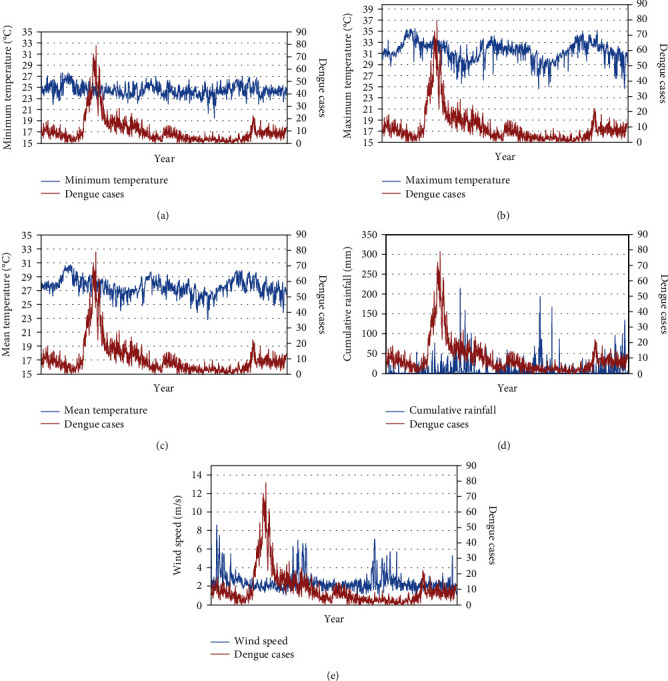
(a) Minimum temperature; (b) maximum temperature; (c) mean temperature; (d) cumulative rainfall; (e) wind speed.

The trend of dengue cases with corresponding weather parameters is illustrated in Figures 1(a)–1(e). A cyclical pattern of dengue cases was observed, with annual peaks coinciding with the intermonsoon season. During this period, the daily maximum temperature was observed to be above 30°C (Figure 1(b)), the daily cumulative rainfall was less than 50 mm (Figure 1(d)), and the average daily wind speed fluctuating around 2.5 m/s (Figure 1(e)) After heavy rainfall, the number of cases was reduced, as depicted in Figure 1(d). We also noted a decline in dengue cases following windy days where the wind speed was more than 5 m/s.

### 3.1. Effect of Maximum Temperature on Dengue Cases in Kelantan

**Figure 2:**
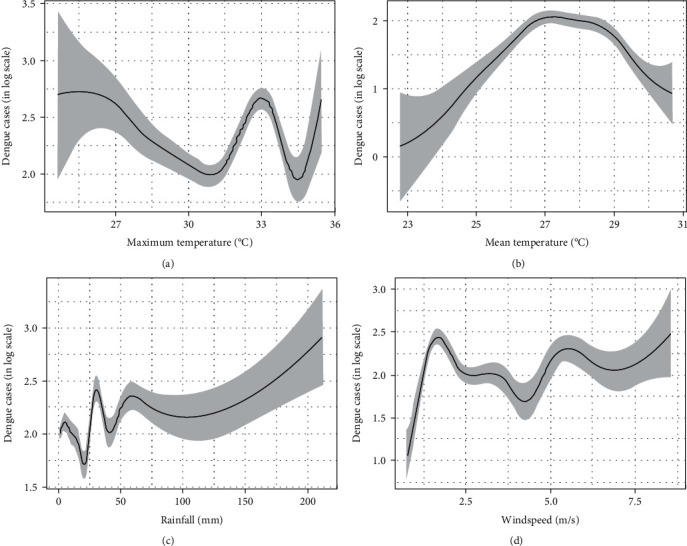
Effect of different weather variables on dengue cases.

The estimated effect of maximum temperature on dengue cases is nonlinear, with a stronger effect between 31.5°C and 34°C (Figure 2(a)). Dengue incidence is estimated to reduce with increased maximum temperature. However, a surge of dengue cases is estimated to cluster around days with a maximum temperature of 33°C, usually coinciding with subsequent heavy rainfall on the following day.

### 3.2. Effect of Mean Temperature on Dengue Cases in Kelantan

The estimated effect of mean temperature on dengue cases is nonlinear with a higher number of dengue cases occurring between 26°C and 28°C (Figure 2(b)). The estimated number of dengue cases rises as the mean temperature rises from 23°C to 26°C. The number of dengue cases is expected to reduce when the daily mean temperature rises above 28°C.

### 3.3. Effect of Rainfall on Dengue Cases in Kelantan

Rainfall has a higher nonlinear effect on dengue when daily cumulative rainfall is below 50 mm per day (Figure 2(c)). The highest number of dengue cases is estimated to occur on days with cumulative daily rainfall between 20 mm and 30 mm. As the cumulative rainfall increases, the effect of rainfall on dengue reduces. Cases of dengue were estimated to increase when the daily cumulative rainfall rises above 100 mm per day.

### 3.4. Effect of Wind Speed on Dengue in Kelantan

In general, a rise in daily wind speed is estimated to increase the number of daily dengue cases (Figure 2(d)). This effect is true with an increase in lower wind speed from 0 m/s to 2 m/s, coinciding with a lower maximum daily temperature. However, a notable reduction in the estimated number of daily dengue cases was observed as the wind speed increased from 2 m/s to 4 m/s.

## 4. Discussion

Our analysis reveals that maximum temperature, mean temperature, rainfall, and wind speed have a significant nonlinear effect on dengue cases in Kelantan. The highest dengue transmission is observed during the intermonsoon period. The intermonsoon period separates the two types of monsoons affecting Malaysia annually, namely, the Southwest Monsoon and the Northeast Monsoon. These periods are characterised by conventional thunderstorms, which are short but intense, accompanied by slower wind speed [19, 20] with high maximum temperature (between 31.5°C and 34°C), high mean temperature (between 23°C and 26°C), low wind speed (between 0 m/s and 2 m/s), and minimal rainfall (between 20 mm and 30 mm). The west coastal region of Peninsular Malaysia experiences less seasonal variation because the Titiwangsa mountain range acts as a buffer to the strong winds and high rainfall associated with the Northeast Monsoon. However, the west coast has greater spatial variability of rainfall compared to the east coast region, with significant dry and wet areas. The east coast region, including Kelantan, sees a more uniform rainfall distribution in all its districts annually [20]. This uniform spatial distribution gives our study an advantage for state-wide analysis by using the Kota Bharu weather station as a proxy. Other studies evaluating the effects of dengue in Malaysia adopted a city- or district-specific approach in consideration of this spatial weather variability [4, 6, 21].

We speculate that the initial dry Southwest Monsoon provides ambient temperature suitable for ovipositing by the female *Aedes* mosquito. Subsequently, the short bursts of rainfall during the intermonsoon period allow the mosquitos to complete their aquatic life cycle and increase the vector density. Effect of the intermonsoon period can also be observed in a study conducted in Kandy City, Sri Lanka, where dengue incidence rises during the second intermonsoon period [13]. This supports the theory that dengue favours days with intermittent rainfall in comparison to days with heavy rainfall.

Compared to the daily maximum and mean temperature, no obvious temporal pattern is observed between the daily minimum with dengue incidence in Kelantan. This observation contradicts previous studies in Malaysia, where the daily minimum temperature was found to have a significant predictive value for dengue incidences [4, 6, 22]. For example, an increase in the daily minimum temperature above 24°C increases the relative risk of dengue in Kuala Lumpur, Selangor, and Putrajaya at a 30-, 60-, and 90-day lag [6]. However, the study area of the previously mentioned studies has a generally higher but narrower range of minimum temperature than Kelantan, which may explain its lack of significance in predicting dengue incidence in Kelantan.

We observed that a lag of one to three months following an increase in maximum or mean temperature coincides with a notable increase in dengue incidences. Although similar observations have been recorded in other studies conducted in Peninsular Malaysia [6], West Malaysia [4], Sri Lanka [13], China [8], and Peru [17], the lag time to observed effect on dengue incidences varies. The variation is primarily attributed to the objective of the respective studies. For example, the incorporation of lag up to 90 days to account for the intrinsic and extrinsic incubation periods of dengue viruses [6] or using a lag of six months to allow for four-month prediction of dengue incidence means that there is adequate time for implementation of mitigative activities [4]. In a study conducted by Mala and Jat [23]. An increase in maximum temperature above 33°C was found to reduce the number of dengue incidences. Temperatures above 30°C have been associated with a decrease in mosquito abundance and survivability, thus affecting the dengue virus transmission [24]. This may explain the reduction in dengue incidence on day 0 when the maximum temperature goes beyond 32°C.

The pattern of dengue case association with temperature can also be explained through viral activity. Virus transmissibility is temperature-dependent during the EIP. At 25°C, the EIP ranges from five days to 33 days. As the temperature rises to 30°C, the EIP reduces to 2.4 days to 15 days [25]. Their findings, however, are based on the daily mean temperature, which is a simplification of the fluctuating temperature witnessed in one day. Temperature fluctuations vary spatially and temporally and are speculated to influence the EIP of dengue viruses. The decrease in EIP means that the time taken by the dengue virus to be transmissible by the *Aedes* mosquito to humans during a blood meal is shorter. The average daily temperature in Kelantan is 27.5°C (SD = 1.2), which theoretically places the dengue EIP in Kelantan to reach up to 33 days. As only 50% of the adult female *Aedes* mosquito is expected to survive beyond 38 days at 27°C [26], some dengue viruses might not be transmissible throughout the mosquito's lifespan. Therefore, an increase in daily temperature favours more dengue virus transmission.

We noted in our study that dengue cases continue to have a positive correlation with an increase in daily cumulative rainfall even beyond 100 mm per day. Although a similar positive correlation was observed in the study by Ehelepola et al. [13], it is noteworthy that the definition of heavy rainfall in Sri Lanka is more than 20 mm a day. This value is five times lower than the cumulative rainfall recorded in the current study. A more comparable effect of rainfall to dengue incidence was observed in a study in Southern Taiwan by Chien and Yu [27]. They reported that the relative risk for dengue increases when the cumulative rainfall increases from 50 mm per day to 130 mm per day but decreases after the 130 mm benchmark at week 0 of rainfall. The relative risk for dengue remains relatively constant at 1.25 up to 15 weeks when controlled for maximum rainfall at 100 mm in 24 hours. Heavy rainfall, noted as the maximum recorded rainfall of more than 330 m within 24 hours, was reported to decrease the incidence of dengue in Southern Taiwan and was speculated to be caused by the washing out of breeding places [7, 12]. Interestingly, there is a discrepancy between the observed trend of rainfall and dengue incidence in Figure 1(d) compared to the association between rainfall and dengue incidence through the general additive model (GAM), which may reflect the limitation of our study. Our analysis is based on the date of onset to reduce the effect of notification bias because it generally takes 0 to three days from symptom onset to seeking medical treatment and subsequent disease notification. However, we are optimistic that the GAM analysis could smoothen out any recall bias by the patients that may be apparent in the rainfall and dengue incidence trend.

According to our findings, an increase in low wind speed has a greater increase in dengue incidences in comparison to a rise in high wind speed. These results are consistent with the findings of Cheong et al. [6] when analysed without any lag between the day of recorded wind speed and the day of recorded dengue cases. We postulate that the effect of the high wind speed in increasing dengue incidence by increasing the vector's flight range is prominent only when dengue transmission is already high in the vicinity. Since there was low dengue incidence at the start of 2018, the increase in wind speed has little effect in increasing dengue transmission. Although our study has the spatial advantage of capturing state-wide dispersed dengue incidences affected by wind, a longer study frame of at least five years may be required to prove this theory. Conversely, higher wind speed has also been reported to reduce dengue incidence by disrupting the mosquito's ability to fly freely and seek a host [6, 22]. Additionally, higher wind speed assists in evaporation formation which, in turn, increases rainfall as deep convection is triggered in the atmospheric boundary layer [20]. Hence, an increase in dengue incidence is caused by an increase in vector breeding sites following an increase in rainfall secondary to high wind speed [7].

Our model did not account for the lag effect between weather variability and its subsequent effect on dengue incidence. We observed previous studies such as the study in Tawau by Jayaraj et al. [4] which used a Seasonal Autoregressive Moving Average (SARIMA) method to test the lag effects between weather variables with dengue. However, this is beyond the scope of our current study. Together with the subjected inherent limitations of secondary data from the passive national dengue surveillance system, including underreporting, biases in reporting, delayed reporting, misreporting, changes in the case definition of dengue, and availability of dengue rapid test kits, only 8.2% of the dengue cases and 14.9% of the deviances in dengue cases occurring in Kelantan are estimated to be explained by our model (*R*^2^ = 0.082).

## 5. Conclusion

Dengue incidences are expected to increase with warmer temperatures, intermittent rainfall, and slow wind speed. We would also like to highlight a local variation where heavy rainfall (cumulative rainfall above 100 mm per day) and high wind speed (an average of more than 7.5 m/s in a day) is linked to high dengue incidence in Kelantan when not accounting for lag effect. Given that weather variables affect nearly 10% of the local dengue incidences, it is relevant to be included as a variable in a dengue early warning system. The use of GAM to visualise the nonlinear relationship of temperature, rainfall, and wind speed can further be generalized to other climates whilst considering seasonality and other weather variables in the model.

Targeted interventions can be recommended, such as hastening vector control by the health authorities before the intermonsoon period starts in April and October. For the control activity to be effective, the buffer zone for vector control activities may have to be expanded up to 1 km on windy days. A collaboration between the Aerodrome Meteorological Office in KLIA under the Malaysian Meteorological Department and the local health authorities by providing an alert of forecasted days with average wind speed above 4 m/s can be initiated. However, the expansion of the buffer zone necessitates a counterbalance between cost-effectiveness and the sheer volume of workload experienced by local health authorities, which can be opportunities for future studies.

## Figures and Tables

**Table 1:** Demography of dengue patients in Kelantan from 2016 to 2018.

Variables	*n* (%)	Mean (SD)
*Total dengue cases*	10,645	
*Demographic*		
*Age (years)*		28.8 (1.23)
≤14	2,481 (23.3)	
15–29	3,801 (35.7)	
29–44	2,140 (20.1)	
45–59	1,498 (14.1)	
≥60	725 (6.8)	
*Gender*		
Male	5,413 (50.9)	
Female	5,232 (49.1)	
*Ethnicity*		
Malay	10,329 (97.0)	
Chinese	222 (2.1)	
Others	94 (0.9)	

**Table 2:** Distribution of dengue cases and selected weather parameters in Kelantan from 2016 to 2018.

Variables	Mean (SD)	Minimum	Percentiles			
			25^th^	50^th^	75^th^	100^th^
Daily total dengue cases	9.8 (11.02)	0	3	7	12	79
Daily minimum temperature (°C)	24.5 (1.05)	19.5	23.8	24.4	25.2	27.6
Daily maximum temperature (°C)	31.4 (1.77)	24.6	30.4	31.5	32.6	35.5
Daily mean temperature (°C)	27.5 (1.20)	22.8	26.8	27.5	28.2	30.7
Daily rainfall (mm)	5.77 (22.83)	0	0.0	0.1	6.1	214.6
Daily wind speed (m/s)	2.4 (0.98)	0.8	1.9	2.2	2.6	8.6

## Data Availability

The aggregated dengue data used to support the findings of this study were supplied by the Ministry of Health Malaysia whereas the weather data was supplied by the Malaysian Meteorological Department. Both datasets are under license and so cannot be made freely available. Requests for access to these data should be made to the Kelantan State Health Department (jknk@moh.gov.my) and the National Climate Centre (klim@met.gov.my).
